# Supplementary material for: Predicting visual function by interpreting a neuronal wiring diagram
Source: Nature. 2024 Oct 2;634(8032):113–23. doi: 10.1038/s41586-024-07953-5 (PMC11446822; doi:10.1038/s41586-024-07953-5)
Supplement: Supplementary file 6 — CRF and ERF predictions for individual TmY4 and TmY9 cells. Analogous to Supplementary Data 3, but for TmY target types. Shown are the top four monosynaptic pathways, the strongest pathway passing through each of the top ten intermediary types (ranking from Extended Data Fig. 7), and the trisynaptic pathway Tm1–TmY–Dm3–TmY (see the section entitled Prediction of spatial normalization). [file 41586_2024_7953_MOESM6_ESM.zip › DataS4/TmY9qΓèÑ/720575940631684293.pdf]

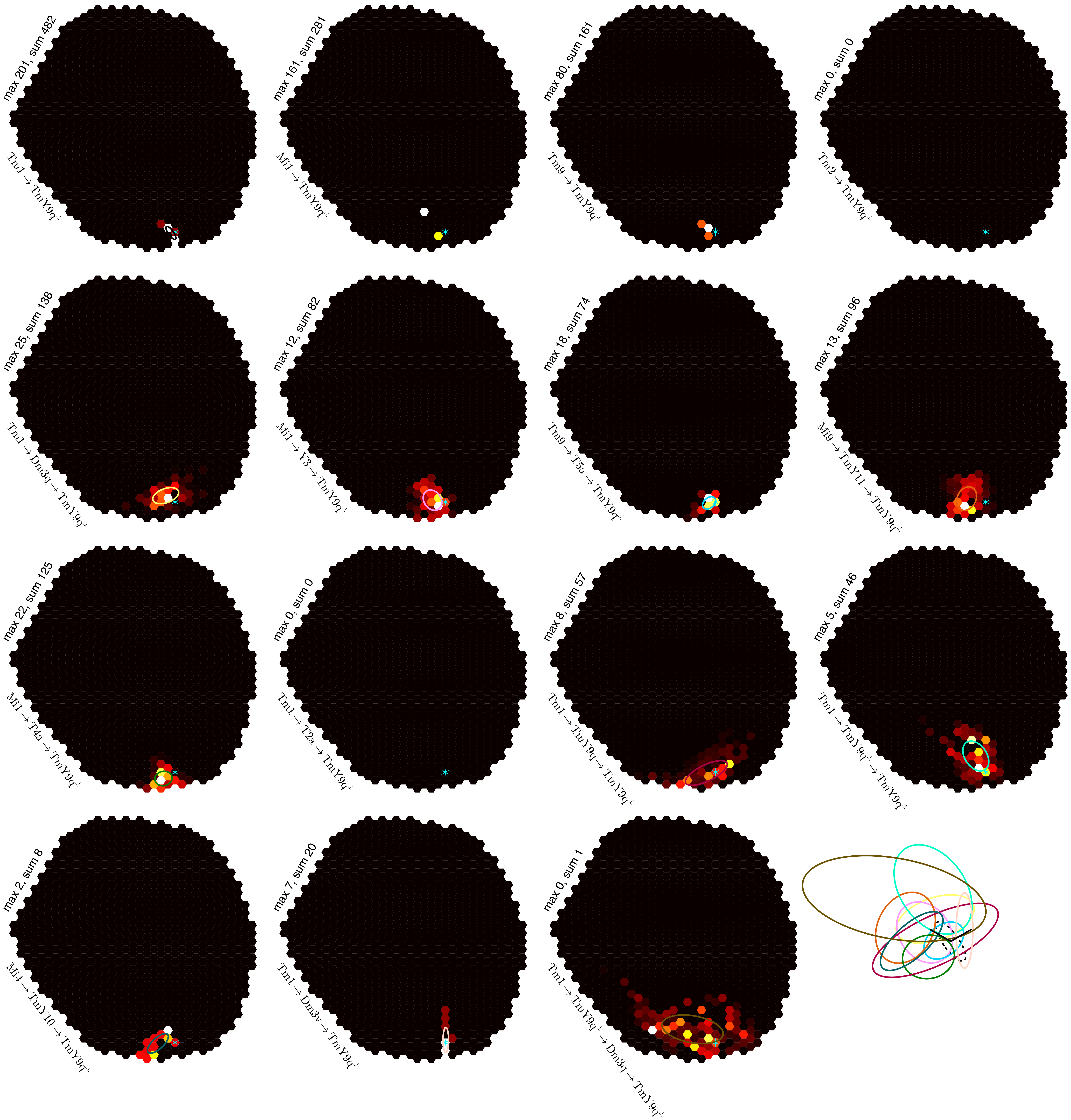

max 201, sum 482
Tm1 → TmY9q⊥
max 161, sum 281
Mi1 → TmY9q⊥
max 80, sum 161
Tm9 → TmY9q⊥
max 0, sum 0
Tm2 → TmY9q⊥
max 25, sum 138
Tm1 → Dm3q → TmY9q⊥
max 12, sum 82
Mi1 → Y3 → TmY9q⊥
max 18, sum 74
Tm9 → T5a → TmY9q⊥
max 13, sum 96
Mi9 → TmY11 → TmY9q⊥
max 22, sum 125
Mi1 → T4a → TmY9q⊥
max 0, sum 0
Tm1 → T2a → TmY9q⊥
max 8, sum 57
Tm1 → TmY9q → TmY9q⊥
max 5, sum 46
Tm1 → TmY9q⊥ → TmY9q⊥
max 2, sum 8
Mi4 → TmY10 → TmY9q⊥
max 7, sum 20
Tm1 → Dm3v → TmY9q⊥
max 0, sum 1
Tm1 → TmY9q⊥ → Dm3q → TmY9q⊥
